# Physical inactivity as risk factor for mortality by diabetes mellitus in Brazil in 1990, 2006, and 2016

**DOI:** 10.1186/s13098-019-0419-9

**Published:** 2019-02-28

**Authors:** Diego Augusto Santos Silva, Mohsen Naghavi, Bruce B. Duncan, Maria Inês Schmidt, Maria de Fatima Marinho de Souza, Deborah Carvalho Malta

**Affiliations:** 1 Research Center in Kinanthropometry and Human Performance, Sports Centre, Postgraduate Program in Physical Education, Federal University of Santa Catarina, University Campus, Trindade, Florianópolis, SC 88010-970 Brazil; 2 Institute for Health Metrics and Evaluation, Seattle, WA USA; 3 Postgraduate Program in Epidemiology, School of Medicine, Federal University of Rio Grande do Sul, Porto Alegre, RS Brazil; 4 Department of Surveillance of Noncommunicable Diseases, and Injuries, and Health Promotion, Ministry of Health, Brasília, DF Brazil; 5 Department of Maternal and Child Nursing and Public Health, School of Nursing, Universidade Federal de Minas Gerais, Belo Horizonte, MG Brazil

**Keywords:** Diabetes, Burden of disease, Hyperglycemia, Mortality, Morbidity, Physical activity

## Abstract

**Background:**

The aims of this study were to estimate the mortality due to diabetes mellitus attributed to physical inactivity in Brazil, to analyze these estimate in three points in time (1990, 2006 and, 2016), and to analyze these estimates according to the socioeconomic status of Brazilian states.

**Methods:**

All deaths and diseases recorded in Brazil during this period were analyzed. Surveys of the general adult population using random sampling procedures evaluating self-reported physical activity in all life domains in Brazil were included. The total number and the age-standardized rates of deaths, and population-attributable fraction (PAF) for diabetes mellitus attributed to physical inactivity in the years 1990, 2006, and 2016 were estimated. Socioeconomic Development Index (SDI) was used as an indicator of socioeconomic status of Brazilian states.

**Results:**

In relation to mortality due to diabetes mellitus attributed to physical inactivity, 736 deaths were estimated in 1990, 1337 deaths were estimated in 2006, and 1897 in 2016, which represented, in 1990, an age-standardized mortality rate (per 100,000 inhabitants) of 1.2, 2.1 in 2006, and 1.1 in 2016. Approximately 3.0% (PAF) of deaths due to diabetes mellitus could be avoided if the Brazilian population were physically active. In 2006 and 2016, Brazilian states with worst socioeconomic indicators had higher age-standardized mortality rate by diabetes mellitus due to physical inactivity.

**Conclusion:**

These findings are the first to estimate the burden of diabetes mellitus due to physical inactivity in Brazil and support the promotion of physical activity in the Brazilian population to prevent and manage diabetes mellitus.

**Electronic supplementary material:**

The online version of this article (10.1186/s13098-019-0419-9) contains supplementary material, which is available to authorized users.

## Background

In 2017, the International Diabetes Federation estimated that there were 451 million (age 18–99 years) people with diabetes worldwide [1]. These figures were expected to increase to 693 million by 2045 [1]. Currently, 70% of people with diabetes live in low- and middle-income countries, and the number of people with diabetes will more than double in these countries over the next 20 years [2, 3]. These estimates are worrying because diabetes was responsible for 294,203 deaths in Brazil between 1996 and 2011 [4].

Diabetes mellitus has multifactorial etiology that includes both genetic and modifiable lifestyle factors [2]. Lifestyle measures, including physical activity, are key factors for prevention and self-management in patients with diabetes to prevent macrovascular complications and premature mortality [3]. Increased physical activity has long been considered a cornerstone of diabetes prevention and management [5]. Dose–response evidence has shown that any type (resistance exercise, aerobic exercise) and intensity (low, moderate and vigorous) in any domain (occupational/domestic, leisure, commuting) of regular physical activity can reduce the onset of diabetes mellitus (type 2, and gestational diabetes) by 25–40% [5–7].

Studies analyzing data on the global burden of mortality and morbidity due to diabetes mellitus have found no association with a single specific cause but with several causal factors of the disease [3, 4, 8–10]. The information on the burden of mortality or morbidity due to diabetes for several causes is important because it provides essential data for epidemiological monitoring of the disease. However, it does not allow identifying and quantifying each risk factor that impact the burden of the disease [10, 11]. The study of physical inactivity as a specific risk factor for mortality and morbidity due to diabetes mellitus may be useful for public policies to intervene not only in relation to the disease itself but also to prioritize intervention policies and greater investment in combating risk factors, which may result, in the medium and long term, in lower incidence of the disease and better quality of life of the affected population [10, 11].

Previous studies in Brazil have shown that there is an inequality in the burden of diabetes mortality among Brazilian states, with regions with lower socioeconomic development showing higher mortality rates due to diabetes [4, 8, 9]. This inequality can be a reflection of coverage and access to the health system, which also presents inequality among regions. In this sense, investigating how the burden of mortality and morbidity due to physical inactivity is growing in Brazilian states over 26 years may be useful to identify whether these inequalities remain in the presence of an important risk factor for diabetes mellitus.

The aims of this study were to estimate the mortality due to diabetes mellitus attributed to physical inactivity in Brazil, to analyze these estimate in three points in time (1990, 2006 and, 2016), and to analyze these estimates according to the socioeconomic status of Brazilian states.

## Methods

### Study overview

The 2016 Global Burden of Disease Study (GBD) includes an annual assessment covering 195 countries and territories from 1990 to 2016. It covered 328 diseases and injuries, 2982 sequelae and 84 risk factors by age and sex. Detailed descriptions of the 2016 GBD methodology and approach have been published elsewhere [10–12].

Data from this study included information on male and female population aged ≥ 25 years. The burden of diabetes in Brazil was evaluated (26 states and Federal District). The total population of Brazil is approximately 200 million inhabitants according to Census [13].

### Diabetes mellitus estimates

Diabetes mellitus in GBD is considered both as a disease and a metabolic risk factor. This study was focused on its burden as a disease. The burden of uncomplicated diabetes, vision loss caused by diabetes (moderate low vision, severe low vision, and blindness), diabetic neuropathy, diabetic foot due to neuropathy, and amputation are included in the burden of diabetes [8, 9].

The International Statistical Classification of Diseases (10th revision, ICD-10) codes related to diabetes mellitus have been mapped. ICD-10 codes for incidence, morbidity and mortality due to diabetes mellitus were E10–E10.1, E10.3–E11.1, E11.3–E12.1, E12.3–E13.1, E13.3–E14.1, E14.3–E14.9, P70.0–P70.2, R73–R73.9 [3, 10]. Additional information about these codes has been previously published [8].

Input data for mortality estimates due to diabetes mellitus in Brazil came from the Vital Registry mortality. For all Brazilian states, the quality of data from the vital registries is considered high and close to high-income countries [14, 15]. Crude data were processed to make them comparable and to account for ‘‘garbage codes’’, which are codes assigned to causes that are not usable from the perspective of public health [16]. These causes were redistributed to the most likely underlying cause of death based on the regression model [12]. Using the Cause of Death Ensemble modeling (CODEm) approach with cause-specific covariates, mortality estimates for each individual cause were computed [12]. These estimates were scaled to fit into an independently modeled all-cause mortality estimate using the CodCorrect algorithm. Since deaths in younger age groups are almost exclusively due to type 1 diabetes, while deaths in older ages are primarily due to type 2 diabetes, two models were used to estimate deaths due to overall diabetes [12]. For this study, we used the second model because we used the information from population aged ≥ 25 years, so the major cause of death in this age group is due to type 2 diabetes rather than due to type 1 diabetes. However, in this model, there were still cases of type 1 diabetes that were not possible to be excluded [11, 12]. In this model, we have added the following covariates to better represent the presented data: education years per-capita of the country and states, a composite score that approximates access to and quality of personal healthcare (Healthcare Access and Quality Index), lag distributed gross domestic product—GDP per capita in base 2010 international dollars, mean diabetes fasting plasma glucose (mmol/L) by age group, age-standardized prevalence of diabetes, estimated national availability of animal fat expressed as kilocalories per capita, age-standardized mean body mass index for adults ages 20+ (separate by sex), mean serum total cholesterol (mmol/L) for individuals above age 25, mean systolic blood pressure (mmHg) for individuals above age 25, estimated energy adjusted national availability of fruits expressed in grams per person per day, estimated energy adjusted national availability of vegetables expressed in grams per person per day, estimated energy adjusted national availability of whole grains expressed in grams per person per day, estimated national availability of dietary energy expressed in kilocalories per person per day [11, 12].

### Physical inactivity estimate

Surveys of the general adult population using random sampling procedures evaluating self-reported physical activity in all life domains (leisure/recreation, work, household and commuting) were included. Due to the absence of a consistent relationship at individual level between the amount of physical activity performed in each domain and total activity, it was not possible to include studies that evaluated only recreational/leisure activities [11].

For global estimates, data were primarily derived from two standardized questionnaires, the Global Physical Activity Questionnaire (GPAQ) and the International Physical Activity Questionnaire (IPAQ), although any other surveys that evaluated intensity, frequency and duration of physical activities performed across all activity domains were included [11].

In the case of Brazil, surveys such the Telephone-based Surveillance of Risk and Protective Factors for Chronic Diseases, Brazil World Health Survey, and the International Prevalence Study on Physical Activity were also consulted [17]. More details can be found at http://ghdx.healthdata.org/gbd-2015/data-input-sources.

To standardize all estimates of physical inactivity in Brazil and around the world, data from the population aged 25 years or more were considered. Reported physical activity was accumulated for durations of at least ten consecutive minutes, across all life domains. Physical activity frequency, duration and intensity were used to calculate the total metabolic equivalent (MET) minutes per week [11, 17]. Estimates included subjects classified as physically inactive (< 600 METS-min/week) [11, 17].

### Analytical methods

The contribution of physical activity for mortality due to diabetes mellitus was estimated using a comparative risk assessment approach in which health outcomes are compared to those that would have been observed with a counterfactual set of exposure where no one was exposed [11]. For this, the simulation model by CODEm was used to estimate indicators by age, sex, country, state, year, and cause, that is, an analytical tool that tests several possible statistical models of causes of death and creates a combined set of models that offers the best predictive performance. The DisMod-MR 2.1 software (World Health Organization©, Geneva, Switzerland), a meta-regression tool, was used for derivation of simultaneous estimates of incidence, prevalence, disability, and mortality, attributed to risk factors, such as physical inactivity [10–12]. We used a recently published dose–response meta-analysis of prospective cohort studies to estimate the effect size of the change in physical activity level on diabetes [18]. Once the raw data had been adjusted to meet our gold standard definition of physical activity, we modeled activity as a single parameter proportion model in DisMod. We estimated the proportion of each country/year/age/sex subpopulation in each of the above four activity levels using six separate Dismod models [10–12]. We use six models rather than four to accommodate the different MET-min/week cutoffs presented in tabulated data sources where individual unit record data was not available (MET-min/week < 600; MET-min/week ≥ 600; MET-min/week 600–3999; MET-min/week > 4000; MET-min/week 4000–7999; MET-min/week ≥ 8000). Since the accepted threshold/definition for inactivity is consistently < 600 MET-min/week, the vast majority of tabulated data was broken down into proportion inactive (model A) and proportion low, moderate or highly active (model B) [10–12]. These models have mesh points at 0, 15, 25, 35, 45, 55, 65, 75, 100, and a study-level fixed effect on integrand variance (Z-cov) for whether a study was nationally representative or not, to account for the heterogeneity introduced by studies that are not generalizable to the entire population. After DisMod, we rescale these 6 models so that the proportions sum to one. Since we have the most data for models A and B, we rescale the sum of the proportion in each category to be equal to one. Next we rescale the sum of model C and D to be equal to the rescaled value from model B. Then we rescale the sum of models E and F to be equal to the rescaled value from model D. After these three rescales we are left with a proportion for each of the four categories that all sum to 1 [10–12]. For the first time, we have directly estimated total MET-min/week globally through the use of a regression that estimated the relationship between total MET-mins/week and each of the categorical prevalence of physical activity. The resultant coefficients were then applied to country–year–age–sex specific estimates of categorical prevalence of physical activity [10–12]. Utilizing microdata on total MET-mins/week from individual-level surveys, we characterized the distribution of activity level at the population level. We then used an ensemble approach to distribution fitting, borrowing characteristics from individual distributions to tailor a unique distribution to fit the data using a weighting scheme. We then applied the coefficients of this regression to the outputs of our estimate of total MET-min/week regression outputs to calculate the standard deviation by country, year, age, and sex. Modeling details can be found in literature [11].

Incident diabetes mellitus cases (Additional file 1), summary exposure value (SEV) to physical inactivity (Additional file 2), absolute number of deaths, mortality rate (per 100,000 inhabitants—crude and age-standardized), and population-attributable fraction (PAF) [11] of death due to diabetes mellitus related to physical inactivity were used as metrics.

The SEV represents a measure of a population’s exposure to a risk factor that takes into account the extent of exposure by risk level and the severity of that risk’s contribution to disease burden. SEV takes the value zero when no excess risk for a population exists and the value one when the population is at the highest level of risk; we report SEV on a scale from 0 to 100% to emphasize that it is risk-weighted prevalence. This measure was standardized by age. Information on the physical inactivity in 1990, 2006, and 2016, by sex, was estimated. More details of SEV are available in the literature [11].

For the PAF estimates [18], we used the information on the systematic review of relevant epidemiological studies. More details about this analysis can be found in the literature [11].

We also analyzed the mortality by diabetes mellitus attributed to physical inactivity according to Socioeconomic Development Index (SDI) of the Brazilian states. The SDI is a summary indicator derived from measures of income per capita, educational attainment, and fertility using the Human Development Index method [10–12]. The SDI has an interpretable scale: zero represents the lowest income per capita, lowest educational attainment, and highest total fertility rate noted across all GBD geographies and one represents the highest income per capita, highest educational attainment, and lowest total fertility rate. SDI values of each Brazilian state in 1990, 2006, and 2016 have been published previously [10].

## Results

In Brazil, 222,988 incident diabetes mellitus cases were estimated in 1990, 369,355 in 2006, and 446,326 in 2016. The information of the incident diabetes mellitus cases by Brazilian state are in Additional file 1. The risk of exposure to physical inactivity of the Brazilian population was 23.10% (95% U.I. 12.75–35.54) in 1990, 23.04% (95% U.I. 12.96–35.15) in 2006, and 23.26% (95% U.I. 12.85–35.75) in 2016. This risk of exposure was similar in 1990, 2006, and 2016. The population of the Brazilian states of the North and Northeast showed slightly greater exposure to physical inactivity in the 3 years analyzed than the Brazilian population in the other regions (Additional file 2).

In relation to mortality due to diabetes mellitus attributed to physical inactivity, 736 deaths were estimated in 1990, 1337 deaths were estimated in 2006, and 1897 in 2016, which represented, in 1990, an age-standardized mortality rate (per 100,000 inhabitants) of 1.2, 2.1 in 2006, and 1.1 in 2016. Table 1 shows information on mortality due to diabetes mellitus attributed to physical inactivity per Brazilian state.

**Table 1:** Number and age-standardized mortality rate (per 100,000 inhabitants) due to diabetes mellitus attributable to physical inactivity around the Brazil, and Brazilian states in 1990, 2006, and 2016 in ages ≥ 25 years

	Mortality due to diabetes mellitus attributable to physical inactivity																		Change^b^ (1990–2006)	Change^b^ (2006–2016)	Change^b^ (1990–2016)
	1990			2006			2016			1990			2006			2016					
	Deaths	95% U.I.		Deaths	95% U.I.		Deaths	95% U.I.		Rate^a^	95% U.I.		Rate^a^	95% U.I.		Rate^a^	95% U.I.				
Brazil	736	165	1322	1337	304	2381	1897	410	3386	1.2	0.3	2.1	1.2	0.2	2.1	1.1	0.2	2.0	0	0	0
Acre	1	0	2	3	1	6	5	1	8	1.0	0.2	1.7	1.3	0.3	2.4	1.2	0.3	2.2	0	0	+
Alagoas	15	3	27	30	7	53	46	9	83	1.7	0.4	3.0	2.0	0.5	3.5	2.2	0.5	3.9	0	0	+
Amapá	1	0	1	2	0	3	4	1	7	0.9	0.2	1.7	1.0	0.3	1.8	1.2	0.3	2.2	0	0	+
Amazonas	5	1	9	12	3	21	23	5	42	1.0	0.2	1.7	1.1	0.3	2.0	1.4	0.3	2.5	0	0	+
Bahia	60	13	109	121	27	214	172	36	314	1.2	0.3	2.2	1.5	0.3	2.6	1.5	0.3	2.7	0	0	+
Ceará	18	04	33	54	12	96	81	17	146	0.7	0.1	1.2	1.1	0.3	2.0	1.2	0.3	2.2	+	0	+
Distrito Federal	5	1	9	10	2	18	15	3	28	1.2	0.3	2.2	1.0	0.2	1.7	0.7	0.2	1.3	0	0	−
Espírito Santo	10	2	18	23	5	41	31	7	58	1.0	0.2	1.7	1.1	0.3	2.0	0.9	0.2	1.7	0	0	0
Goiás	12	2	22	28	6	50	47	10	85	0.9	0.2	1.6	1.0	0.2	1.7	1.0	0.2	1.8	0	0	0
Maranhão	18	4	33	49	12	87	72	15	129	1.1	0.2	1.9	1.6	0.4	2.9	1.7	0.4	3.1	+	0	+
Mato Grosso	5	1	9	15	3	27	27	5	49	0.9	0.2	1.6	1.3	0.3	2.2	1.3	0.3	2.4	0	0	+
Mato Grosso do Sul	5	1	9	13	3	23	19	4	35	0.8	0.2	1.5	1.0	0.2	1.8	1.0	0.2	1.8	0	0	0
Minas Gerais	76	16	137	119	27	211	167	39	307	1.1	0.2	1.9	0.9	0.2	1.6	0.8	0.2	1.5	0	0	−
Paraná	33	7	59	73	17	128	112	26	203	1.0	0.2	1.7	1.2	0.3	2.0	1.1	0.3	2.0	0	0	0
Paraíba	21	5	38	43	10	75	59	12	106	1.4	0.3	2.5	1.7	0.4	3.1	1.8	0.4	3.3	0	0	+
Pará	11	2	20	32	7	59	60	12	110	0.7	0.2	1.3	1.2	0.3	2.1	1.4	0.3	2.6	+	0	+
Pernambuco	51	11	94	96	23	173	120	26	216	1.6	0.4	2.9	1.9	0.4	3.5	1.8	0.4	3.1	0	0	0
Piaui	7	1	12	23	5	41	36	8	66	0.7	0.2	1.2	1.3	0.3	2.4	1.6	0.4	2.9	+	0	+
Rio de Janeiro	121	28	218	184	41	331	223	50	395	1.7	0.4	3.1	1.5	0.3	2.8	1.3	0.3	2.3	0	0	−
Rio Grande do Norte	13	3	23	28	7	51	46	10	82	1.1	0.2	2.0	1.5	0.3	2.6	1.7	0.4	3.0	0	0	+
Rio Grande do Sul	41	9	74	79	18	141	112	23	203	0.9	0.2	1.6	1.0	0.2	1.7	0.9	0.2	1.6	0	0	0
Rondônia	3	1	5	8	2	14	12	3	23	1.2	0.3	2.2	1.4	0.3	2.4	1.4	0.3	2.5	0	0	0
Roraima	1	0	1	2	0	4	3	1	6	1.7	0.4	3.1	1.8	0.4	3.2	1.9	0.4	3.4	0	0	0
Santa Catarina	18	4	33	35	8	63	55	10	98	1.1	0.2	2.0	1.0	0.3	1.9	1.0	0.2	1.7	0	0	0
Sergipe	11	3	20	19	5	34	28	6	51	1.9	0.4	3.4	2.0	0.5	3.5	2.0	0.4	3.7	0	0	0
São Paulo	170	37	304	231	54	415	308	64	559	1.2	0.3	2.2	0.9	0.2	1.6	0.8	0.2	1.4	0	0	−
Tocantins	2	0	4	7	2	13	13	3	23	0.8	0.2	1.4	1.2	0.3	2.1	1.4	0.3	2.5	+	0	+

U.I.: uncertainty interval; 0: the change in age-standardized mortality rate (per 100,000 inhabitants) was stable in the years analyzed; +: there was an increase in the age-standardized mortality rate (per 100,000 inhabitants) in the analyzed years; −: there was an decrease in the age-standardized mortality rate (per 100,000 inhabitants) in the analyzed years

^a^Age-standardized rate

^b^Change in age-standardized mortality rate due to diabetes mellitus attributable to physical inactivity

**Fig. 1:**
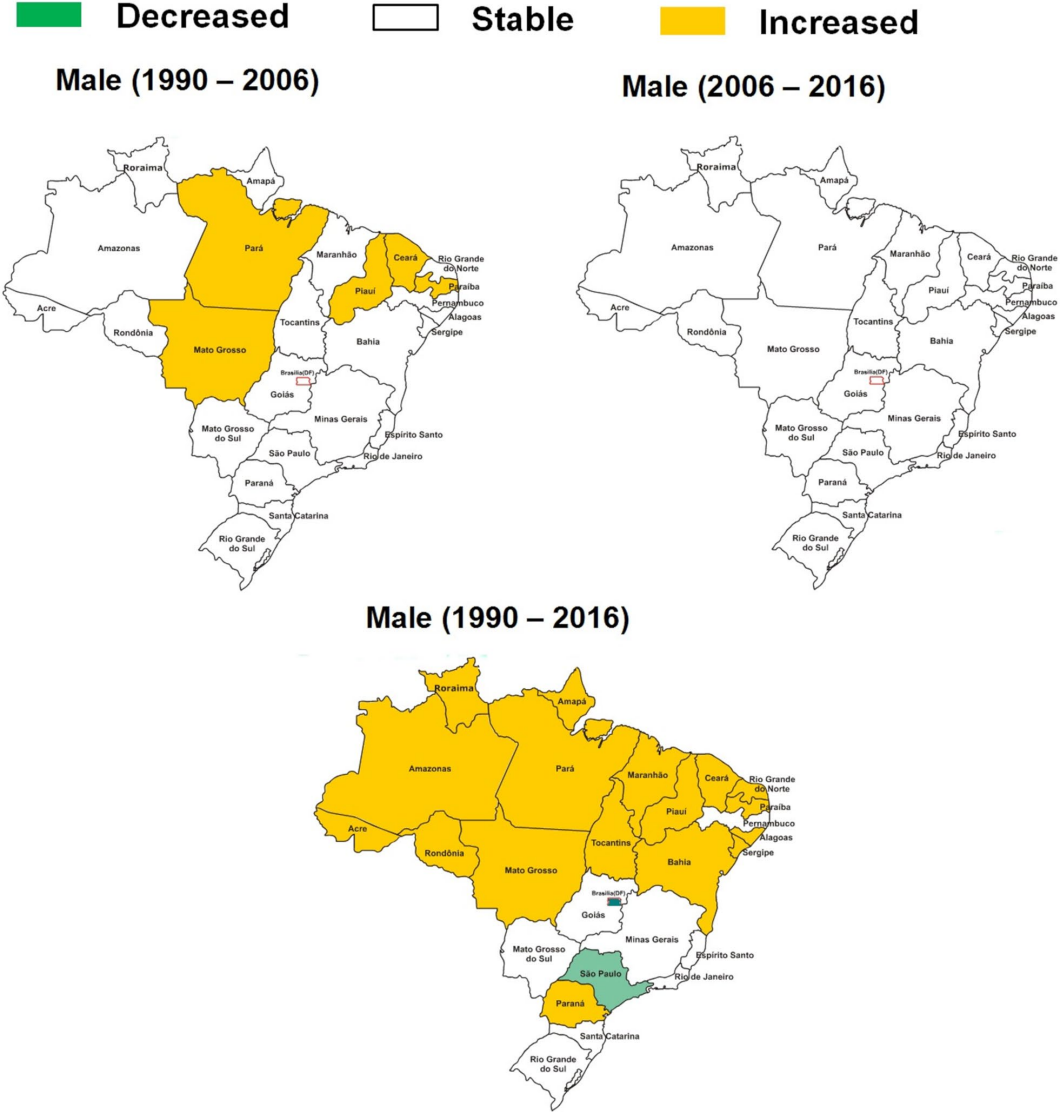
Change in age-standardized mortality rate (per 100,000 inhabitants) due to diabetes mellitus attributable to physical inactivity in men (≥ 25 years old) from Brazil (1990–2006; 2006–2016; and 1990–2016)

**Fig. 2:**
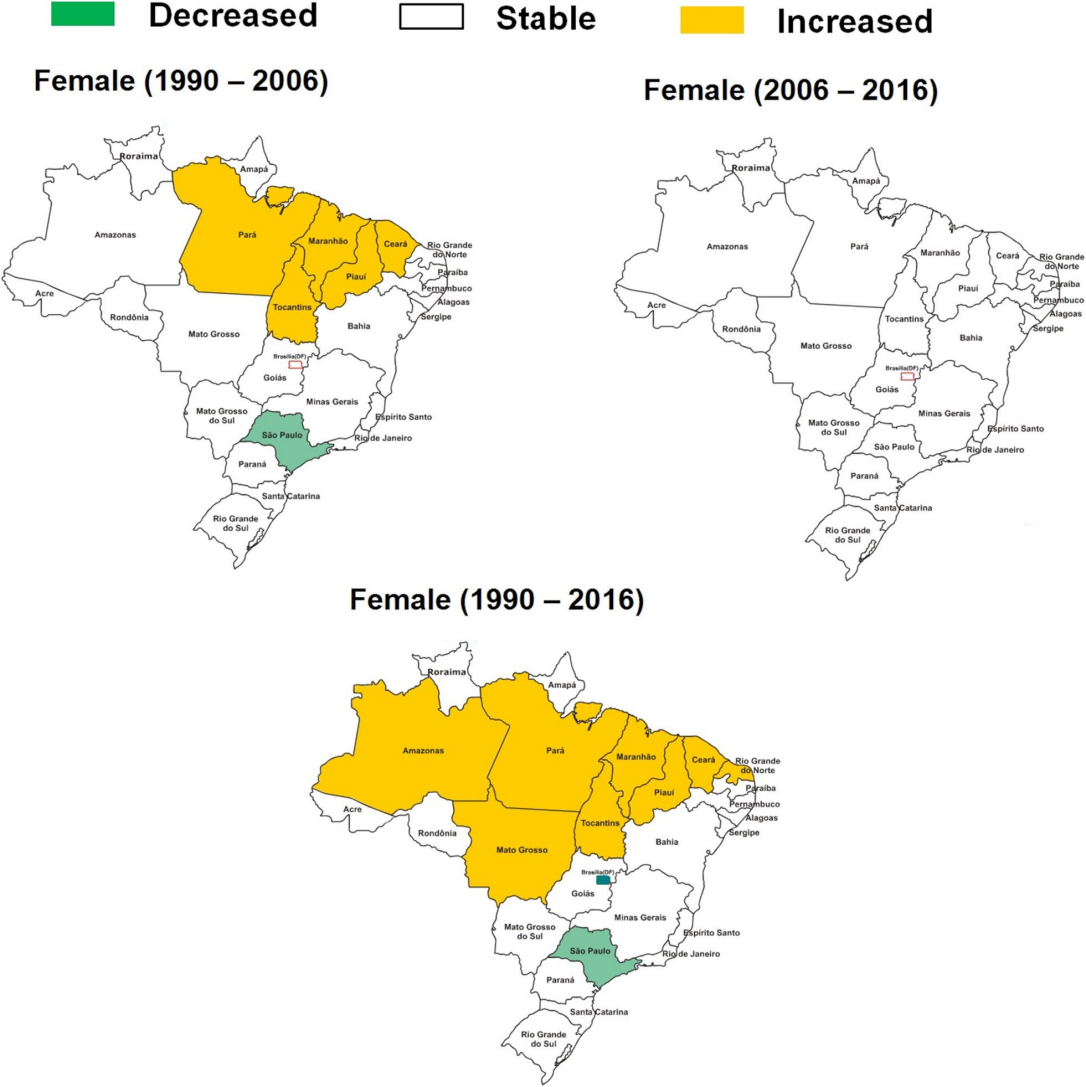
Change in age-standardized mortality rate (per 100,000 inhabitants) due to diabetes mellitus attributable to physical inactivity in women (≥ 25 years old) from Brazil (1990–2006; 2006–2016; and 1990–2016)

From 1990 to 2006, there was stability in age-standardized mortality rate due to diabetes mellitus attributed to physical inactivity in Brazil (0.02%; 95% U.I. − 0.28 to 0.42). However, in the Brazilian states of Ceará, Maranhão, Pará, Piauí, and Tocantins there was an increase in age-standardized mortality rate due to diabetes mellitus attributed to physical inactivity. From 2006 to 2016, there was stability in age-standardized mortality rate due to diabetes mellitus attributed to physical inactivity in Brazil (− 0.08%; 95% U.I. − 0.33 to 0.30), and in all Brazilian states. From 1990 to 2016, there was stability in age-standardized mortality rate due to diabetes mellitus attributed to physical inactivity in Brazil (− 0.06%; 95% U.I. − 0.10 to 0.00). However, in the some states of Noth of Brazil (Acre, Amapá, Amazonas, Pará, and Tocantins), Northeast (Alagoas, Bahia, Ceará, Maranhão, Paraíba, Piauí, and Rio Grande do Norte), and Midwest (Mato Grosso), there was an increase in age-standardized mortality rate due to diabetes mellitus attributed to physical inactivity. From 1990 to 2016, there was an decreased in age-standardized mortality rate due to diabetes mellitus attributed to physical inactivity in the Federal District of Brazil (Midwest geographic region), Minas Gerais (Southeast geographic region), Rio de Janeiro (Southeast geographic region), and São Paulo (Southeast geographic region) (Table 1). These results were similar for men (Fig. 1) and women (Fig. 2) in Brazil.

The age-standardized mortality rate (per 100,000 inhabitants) due to diabetes mellitus attributable to physical inactivity was higher at the more advanced ages compared to the younger. Approximately 3.0% (PAF) of deaths due to diabetes mellitus could be avoided if the Brazilian population were physically active (Table 2).

**Table 2:** Mortality rate (per 100,000 inhabitants) due to diabetes mellitus attributable to physical inactivity, and population attributable fraction in Brazil according to age in 1990, 2006, and 2016

	Mortality due to diabetes mellitus attributable to physical inactivity	
	Rate^a^ (95% U.I.)	PAF: % (95% U.I.)
1990		
Male		
25–49 years	0.1 (0.0–0.2)	2.8 (0.6–5.1)
50–69 years	1.8 (0.4–3.2)	3.2 (0.7–5.7)
70+ years	8.5 (2.0–15.2)	3.4 (0.7–6.0)
Female		
25–49 years	0.1 (0.0–0.2)	2.8 (0.6–5.0)
50–69 years	2.1 (0.5–3.8)	3.3 (0.7–5.9)
70+ years	10.9 (2.5–19.5)	3.5 (0.8–6.1)
2006		
Male		
25–49 years	0.1 (0.0–0.2)	2.9 (0.7–5.1)
50–69 years	1.8 (0.4–3.2)	3.1 (0.8–6.0)
70+ years	10.0 (2.3–17.6)	3.4 (0.8–6.0)
Female		
25–49 years	0.1 (0.0–0.1)	2.9 (0.7–5.2)
50–69 years	1.8 (0.4–3.1)	3.3 (0.8–5.8)
70+ years	11.4 (2.6–20.1)	3.4 (0.8–6.1)
2016		
Male		
25–49 years	0.1 (0.0–0.2)	2.9 (0.7–5.3)
50–69 years	1.8 (0.4–3.3)	3.2 (0.7–5.8)
70+ years	10.2 (2.2–18.2)	3.4 (0.8–6.1)
Female		
25–49 years	0.1 (0.0–0.2)	3.0 (0.7–5.4)
50–69 years	1.6 (0.4–2.9)	3.3 (0.7–5.9)
70+ years	11.0 (2.4–19.5)	3.5 (0.8–6.2)

PAF: population attributable fraction; U.I.: uncertainty interval

^a^Rate per 100,000 inhabitants

**Fig. 3:**
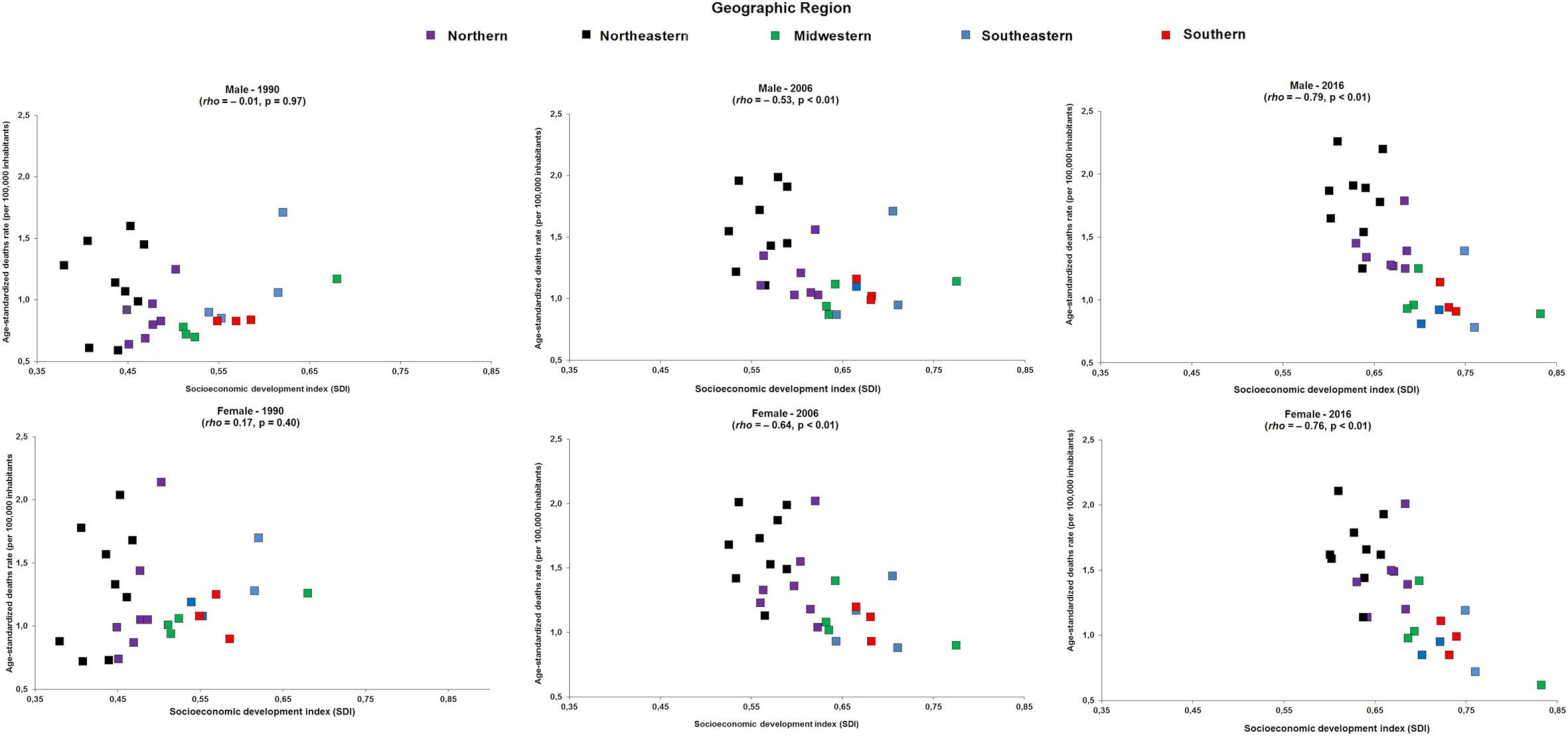
Relationship between age-standardized mortality rate (per 100,000 inhabitants) due to diabetes mellitus attributable to physical inactivity and socioeconomic development index (SDI) in men and women (≥ 25 years old) from Brazil in 1990, 2006 and 2016 according to Brazilian geographic region. rho: Spearman’s correlation coefficient

In 1990, there was no association between the socioeconomic indicator of Brazilian states with age-standardized mortality rate due to diabetes mellitus attributed to physical inactivity in men [Spearman’s correlation coefficient (*rho*) = − 0.01, p-value = 0.97)], and women (*rho* = 0.17, p-value = 0.40). In 2006, the Brazilian states with the worst socioeconomic indicators had higher age-standardized mortality rate due to diabetes mellitus attributed to physical inactivity in men (*rho* = − 0.53, p-value < 0.01). and women (*rho* = − 0.64, p-value < 0.01), and in 2016 this association was of greater magnitude in men (*rho* = − 0.79, p-value < 0.01) and women (*rho* = − 0.76, p-value < 0.01), which revealed a higher age-standardized mortality rate due to diabetes mellitus attributed to physical inactivity in the Brazilian states with the worst socioeconomic indicators (Fig. 3).

## Discussion

Among the main findings of this study, it was found that from 1990 to 2016 the estimates of incidence cases from diabetes mellitus were stable in Brazil. From 1990 to 2016, there were a series of actions in the health policies of Brazil that can justify this stability. In early 1990s, the Ministry of Health of Brazil launched a National Diabetes Plan, which proposed an equation to calculate the amount of insulin needed per year in Brazil for universal coverage and amplified public purchase of the drug [19]. Throughout the 1990s, the restructuring of care for chronic conditions such as hypertension and diabetes at the primary care level progressed, with increasing free public distribution of low cost antidiabetic medications [19]. In 2001, the Plan to Reorganize Care of Hypertension and Diabetes Mellitus [20], was launched, which emphasized the universal treatment of these conditions at primary care level. In 2002, the National Program of Pharmaceutical Provision for Hypertension and Diabetes was created, which allowed a progressively larger and free distribution of medicines and medical supplies to the entire population with diabetes in Brazil [20]. In 2011, the Strategic Action Plan for Confronting Chronic Non-communicable Diseases in Brazil 2011–2022 was created, which proposed actions to combat diabetes mellitus at the population level [21].

The present study reveals that physical inactivity is a significant risk factor for mortality due to diabetes mellitus and can avoid, approximately 3.0–3.5% of mortality cases due to diabetes mellitus in Brazil in all ages. In this sense, actions to promote physical activity for the population can join the aforementioned factors [19] to reduce the burden of diabetes in Brazil. However, some initiatives developed in Brazil have focused on the promotion of physical activity, such as the National Health Promotion Policy [22], the creation of community physical activity programs and the inclusion of physical activity in health monitoring and surveillance systems [23], it is estimated that 62% of the Brazilian adult population is insufficiently active in leisure time and these estimates increase with advancing age [24]. This situation can partially explain the increase in mortality due to diabetes mellitus attributed to physical inactivity with advancing age found in this study.

The other finding of this study was that the age-standardized mortality rate due to diabetes mellitus attributed to physical inactivity remained stable in Brazil from 1990 to 2016 (1990–2006, and 2006–2016). More specific actions to promote physical activity in the Brazilian population are necessary to cause declines in the mortality in the coming years. Among these actions, it is necessary a restructuring of macrostructural factors, such as adapting the environment of cities with the construction of squares and bicycle paths that allow the population to feel motivated to practice physical activity [25]. Brazilian researchers have shown that air pollution, few green areas and poor quality of urban infrastructure are still a challenge in Brazilian cities [26].

Several biological mechanisms could explain an inverse association between physical activity and diabetes mellitus burden. Physical activity improves energy balance and reduces adiposity, which is the main risk factor for diabetes [27]. Physical activity improves glucose homeostasis by increasing skeletal muscle glucose uptake by translocation of GLUT4 glucose transporters to the skeletal muscle cell membranes and by increased activity of glycogen synthase, which may contribute to reduce blood glucose levels [28]. Physical activity increases the secretion of interleukin-6 (IL-6) from muscle cells, which has anti-inflammatory effects through inhibition of TNF-a and IL-1b, and reduces TNF-induced insulin resistance [29]. The American College of Sports Medicine and the American Diabetes Association considers that physical activity has an A level of evidence (highest level of certainty) to prevent diabetes and to assist in treating individuals with the disease at all ages [30]. In this sense, avoiding physical inactivity can be a way to reduce the burden of diabetes mellitus in Brazil and around the world.

The present study revealed major inequalities in the composition of mortality and from diabetes mellitus due to physical inactivity according to Brazilian states. The change in the mortality from diabetes mellitus attributed to physical inactivity was higher in Brazilian states with worse socioeconomic conditions (Northern and Northeastern regions). These states had an increase in mortality in 2006 compared to 1990, and in 2016 compared to 1990. These inequalities reflect a set of structural disadvantages and aspects of social organization in cities that influence the patterns of behavior and ways of life and work, generating uneven exposure to risk and protection factors among Brazilian population, thereby influencing their life-course trajectories and disproportionate risk of death [27]. Brazil is a country of great inequalities, and its national health system has established the goal to diminish those seen in health. At regional level, the Northern and Northeastern regions are considerably poorer than Brazil as a whole. Traditionally residents of this region have had less access to health care [19], and according to the results of the present study they are more exposed to physical inactivity than residents of other regions of Brazil (Additional file 2), and this fact can explain the findings of the present study.

It was found that from 1990 to 2016, age-standardized mortality rate (per 100,000 inhabitants) due to diabetes mellitus attributable to physical inactivity remained stable in men, while in women, it had a decrease. An explanation for this better scenario in women may be that the prevalence of sufficient physical activity during leisure time among Brazilian women increased more than in men over the last decade [28]. In addition, men have higher waist-to height ratio, greater amounts of visceral and hepatic fat and are more insulin resistant than women [29, 30]. In addition, women use health care services more frequently than men [31].

This study has important limitations. First, this study did not stratified diabetes mellitus according to its different types (insulin-dependent, noninsulin-dependent, neonatal) to identify which has greater relation with physical inactivity [32, 33]. Second, the present study included only physical activity measured by questionnaires that are considered subjective measures of physical activity and associated with measurement bias [33]. Third, the non-stratification of physical activity by domains is another limitation. Fourth, the use of only three points in time (1990, 2006, and 2016) to make comparisons. Fifth the use of a simulation model may be considered another limitation, although this model has evidence of validity [10–12].

The strength of this study was the estimation of mortality by physical inactivity throughout Brazil. From this information, it was possible to estimate mortality rates due to diabetes mellitus attributable to physical inactivity. This is the first study that used data from all states to make these estimates, which increases the information accuracy and resolution. Until now, it was known that physical inactivity was a risk factor for diabetes mellitus, but it had not been estimated how much it was related in the three points in time in the same country.

It could be concluded that physical inactivity has contributed to a substantial number of deaths due to diabetes mellitus in Brazil and the Brazilian states. Estimates of mortality from diabetes mellitus as a result of physical inactivity in Brazil were stable between 1990 and 2016. However, the Brazilian states with worse socioeconomic indicators had higher mortality rates due to diabetes mellitus as a result of physical inactivity. These findings support the promotion of physical activity in the Brazilian population to help prevent and manage diabetes mellitus.

## Additional files

**Additional file 1.** Incident cases from diabetes mellitus in Brazil, and Brazilian states in 1990, 2006, and 2016.

**Additional file 2.** Summary exposure value for physical inactivity in Brazil, and Brazilian states in 1990, 2006, and 2016.
