# Supplementary material for: Physical inactivity as risk factor for mortality by diabetes mellitus in Brazil in 1990, 2006, and 2016
Source: Diabetol Metab Syndr. 2019 Feb 28;11:23. doi: 10.1186/s13098-019-0419-9 (PMC6396532; doi:10.1186/s13098-019-0419-9)
Supplement: Supplementary file 1 — Additional file 1. Incident cases from diabetes mellitus in Brazil, and Brazilian states in 1990, 2006, and 2016. [file 13098_2019_419_MOESM1_ESM.docx]

**Additional Digital Content 1.** Incident cases from diabetes mellitus in Brazil, and Brazilian states in 1990, 2006, and 2016.

|  | **Diabetes mellitus incidence** | | | | | | | | | | | | | | | | | | | | |
| --- | --- | --- | --- | --- | --- | --- | --- | --- | --- | --- | --- | --- | --- | --- | --- | --- | --- | --- | --- | --- | --- |
|  | **1990** |  |  | **2006** |  |  | **2016** |  |  | **1990** |  |  | **2006** |  |  | | | **2016** | |  |  |
|  | **Number** | **95% U.I.** | | **Number** | **95% U.I.** | | **Number** | **95% U.I.** | | **Rate*** | **95% U.I.** | | **Rate*** | **95% U.I.** | | | **Rate*** | | **95% U.I.** | | |
| Brazil | 222,988 | 203,028 | 244,612 | 369,355 | 336,113 | 405,932 | 446,326 | 400,340 | 495,708 | 195.6 | 178.5 | 213.7 | 222.7 | 202.9 | | 243.1 | | 211.7 | | 189.4 | 234.3 |
| Acre | 496 | 445 | 554 | 1,015 | 889 | 1,143 | 1,357 | 1,196 | 1,538 | 172.6 | 155.4 | 190.2 | 222.6 | 195.6 | | 248.3 | | 212.4 | | 187.2 | 238.3 |
| Alagoas | 4,038 | 3,654 | 4,497 | 6,921 | 6,269 | 7,728 | 8,716 | 7,783 | 9,726 | 233.3 | 211.4 | 258.6 | 292.0 | 263.5 | | 324.8 | | 294.2 | | 262.6 | 327.5 |
| Amapá | 319 | 285 | 357 | 812 | 725 | 921 | 1,233 | 1,091 | 1,392 | 172.5 | 154.3 | 190.4 | 205.3 | 184.3 | | 228.8 | | 203.3 | | 181.2 | 227.3 |
| Amazonas | 2,204 | 1,939 | 2,479 | 4,67 | 4,174 | 5,265 | 6,808 | 6,050 | 7,676 | 168.0 | 149.3 | 187.2 | 212.5 | 189.5 | | 236.1 | | 219.2 | | 195.7 | 245.7 |
| Bahia | 16,235 | 14,673 | 18,090 | 29,461 | 26,211 | 32,486 | 36,131 | 32,146 | 40,502 | 188.9 | 171.5 | 208.7 | 248.9 | 222.4 | | 272.8 | | 245.2 | | 218.4 | 274.2 |
| Ceará | 6,375 | 5,619 | 7,120 | 13,482 | 11,998 | 14,966 | 16,932 | 15,036 | 19,030 | 136.3 | 120.6 | 151.4 | 199.9 | 178.6 | | 220.9 | | 200.2 | | 177.8 | 225.1 |
| Distrito Federal | 2,514 | 2,223 | 2,808 | 4,393 | 3,884 | 4,993 | 5,718 | 5,045 | 6,484 | 217.7 | 193.9 | 240.9 | 221.9 | 196.8 | | 247.2 | | 192.6 | | 169.5 | 218.0 |
| Espírito Santo | 3,666 | 3,277 | 4,082 | 6,909 | 6,148 | 7,729 | 8,238 | 7,398 | 9,257 | 182.3 | 162.6 | 201.2 | 219.5 | 196.3 | | 244.2 | | 198.1 | | 178.3 | 222.0 |
| Goiás | 5,200 | 4,681 | 5,773 | 9,791 | 8,718 | 10,911 | 13,041 | 11,624 | 14,582 | 172.7 | 157.2 | 190.8 | 195.9 | 175.1 | | 216.8 | | 193.6 | | 173.0 | 215.4 |
| Maranhão | 5,777 | 5,217 | 6,379 | 11,515 | 10,405 | 12,742 | 14,882 | 13,395 | 16,528 | 176.0 | 158.8 | 195.5 | 250.5 | 226.0 | | 276.8 | | 257.6 | | 230.5 | 285.2 |
| Mato Grosso | 2,399 | 2,131 | 2,663 | 5,395 | 4,82 | 6,089 | 7,182 | 6,352 | 8,079 | 171.2 | 153.6 | 190.6 | 230.5 | 207.6 | | 257.2 | | 225.9 | | 200.8 | 250.9 |
| Mato Grosso do Sul | 2,357 | 2,119 | 2,617 | 4,247 | 3,808 | 4,755 | 5,396 | 4,836 | 6,046 | 175.2 | 157.5 | 194.3 | 209.1 | 187.3 | | 233.0 | | 202.5 | | 181.5 | 225.9 |
| Minas Gerais | 23,419 | 21,145 | 25,955 | 33,663 | 30,519 | 36,834 | 41,100 | 36,284 | 46,267 | 187.3 | 169.4 | 208.8 | 183.7 | 166.2 | | 201.2 | | 181.1 | | 160.1 | 202.8 |
| Paraná | 11,877 | 10,715 | 13,386 | 21,245 | 18,895 | 24,057 | 25,636 | 22,621 | 28,863 | 177.5 | 159.5 | 199.4 | 223.6 | 200.9 | | 250.0 | | 212.5 | | 189.0 | 237.7 |
| Paraíba | 4,731 | 4,246 | 5,243 | 8,459 | 7,563 | 9,571 | 10,225 | 9,094 | 11,489 | 207.4 | 186.5 | 230.6 | 270.1 | 241.7 | | 306.5 | | 266.2 | | 237.0 | 297.6 |
| Pará | 4,690 | 4,167 | 5,284 | 10,477 | 9,282 | 11,74 | 14,909 | 13,247 | 16,892 | 137.0 | 121.5 | 153.9 | 203.7 | 181.5 | | 226.4 | | 217.3 | | 194.1 | 245.7 |
| Pernambuco | 11,884 | 10,859 | 13,157 | 20,393 | 18,288 | 22,563 | 23,402 | 20,639 | 26,467 | 223.2 | 204.8 | 246.2 | 285.0 | 255.6 | | 316.8 | | 263.1 | | 232.3 | 297.7 |
| Piaui | 2,482 | 2,211 | 2,764 | 5,286 | 4,758 | 5,871 | 6,869 | 6,121 | 7,767 | 136.8 | 121.9 | 151.9 | 210.3 | 190.2 | | 232.7 | | 227.8 | | 204.5 | 256.2 |
| Rio de Janeiro | 29,432 | 26,684 | 32,506 | 42,567 | 38,093 | 47,155 | 45,586 | 40,510 | 51,726 | 255.2 | 232.1 | 280.7 | 270.9 | 243.6 | | 297.1 | | 241.3 | | 215.6 | 271.4 |
| Rio Grande do Norte | 3,285 | 2,985 | 3,636 | 6,43 | 5,789 | 7,017 | 8,492 | 7,538 | 9,598 | 186.0 | 169.6 | 206.0 | 247.5 | 222.8 | | 270.7 | | 252.8 | | 223.9 | 284.8 |
| Rio Grande do Sul | 13,210 | 11,697 | 14,733 | 21,121 | 18,793 | 23,737 | 24,736 | 21,781 | 27,965 | 167.7 | 148.7 | 185.4 | 195.0 | 174.0 | | 217.5 | | 188.5 | | 167.1 | 211.8 |
| Rondônia | 1,397 | 1,243 | 1,563 | 2,799 | 2,481 | 3,162 | 3,600 | 3,183 | 4,050 | 198.9 | 176.5 | 221.4 | 238.0 | 212.9 | | 264.6 | | 224.3 | | 201.6 | 250.1 |
| Roraima | 291 | 257 | 332 | 750 | 672 | 844 | 1,061 | 939 | 1,205 | 244.4 | 216.9 | 274.2 | 282.1 | 252.3 | | 313.5 | | 270.6 | | 240.9 | 307.8 |
| Santa Catarina | 6,146 | 5,478 | 6,910 | 11,166 | 9,938 | 12,548 | 13,992 | 12,353 | 15,876 | 181.7 | 162.9 | 202.4 | 205.4 | 183.7 | | 228.0 | | 191.7 | | 170.3 | 215.3 |
| Sergipe | 2,666 | 2,409 | 2,950 | 4,631 | 4,111 | 5,155 | 5,850 | 5,163 | 6,675 | 260.0 | 233.9 | 288.9 | 302.1 | 268.3 | | 335.8 | | 289.4 | | 254.9 | 328.7 |
| São Paulo | 54,934 | 49,071 | 61,524 | 79,573 | 71,814 | 89,199 | 92,222 | 81,341 | 103,238 | 208.2 | 187.1 | 231.3 | 206.3 | 186.0 | | 230.4 | | 188.8 | | 167.4 | 209.6 |
| Tocantins | 965 | 864 | 1,071 | 2,17 | 1,937 | 2,419 | 3,010 | 2,688 | 3,378 | 160.2 | 143.5 | 177.4 | 214.1 | 191.2 | | 236.8 | | 222.7 | | 199.0 | 249.1 |

*Age-standardized rate (per 100,000 inhabitants); U.I.: uncertainty interval.
