# Supplementary material for: Physical inactivity as risk factor for mortality by diabetes mellitus in Brazil in 1990, 2006, and 2016
Source: Diabetol Metab Syndr. 2019 Feb 28;11:23. doi: 10.1186/s13098-019-0419-9 (PMC6396532; doi:10.1186/s13098-019-0419-9)
Supplement: Supplementary file 2 — Additional file 2. Summary exposure value for physical inactivity in Brazil, and Brazilian states in 1990, 2006, and 2016. [file 13098_2019_419_MOESM2_ESM.docx]

**Additional Digital Content 2.** Summary exposure value for physical inactivity in Brazil, and Brazilian states in 1990, 2006, and 2016.

|  | **1990** |  |  | **2006** |  |  | **2016** |  |  |
| --- | --- | --- | --- | --- | --- | --- | --- | --- | --- |
|  | **SEV - %*** | **95% U.I.** | | **SEV - %*** | **95% U.I.** | | **SEV - %*** | **95% U.I.** | |
| Brazil | 23.10 | 12.75 | 35.54 | 23.04 | 12.96 | 35.15 | 23.26 | 12.85 | 35.75 |
| Acre | 24.25 | 13.43 | 37.15 | 24.08 | 13.57 | 36.71 | 24.24 | 13.40 | 37.06 |
| Alagoas | 23.57 | 13.02 | 36.20 | 23.48 | 13.23 | 35.83 | 23.68 | 13.12 | 36.21 |
| Amapá | 23.80 | 13.16 | 36.48 | 23.71 | 13.36 | 36.10 | 23.84 | 13.16 | 36.53 |
| Amazonas | 24.01 | 13.28 | 36.75 | 23.82 | 13.43 | 36.37 | 24.07 | 13.30 | 36.78 |
| Bahia | 23.27 | 12.80 | 35.86 | 23.19 | 13.04 | 35.41 | 23.35 | 12.91 | 35.82 |
| Ceará | 23.56 | 13.00 | 36.12 | 23.47 | 13.21 | 35.75 | 23.61 | 13.03 | 36.25 |
| Distrito Federal | 23.10 | 12.73 | 35.56 | 23.02 | 12.96 | 35.18 | 23.16 | 12.81 | 35.62 |
| Espírito Santo | 22.99 | 12.68 | 35.42 | 22.91 | 12.90 | 35.05 | 23.19 | 12.81 | 35.74 |
| Goiás | 23.44 | 12.96 | 35.95 | 23.34 | 13.12 | 35.63 | 23.47 | 12.95 | 35.87 |
| Maranhão | 23.58 | 12.98 | 36.24 | 23.45 | 13.20 | 35.77 | 23.75 | 13.12 | 36.45 |
| Mato Grosso | 23.57 | 13.01 | 36.12 | 23.49 | 13.25 | 35.86 | 23.72 | 13.11 | 36.35 |
| Mato Grosso do Sul | 22.65 | 12.49 | 34.93 | 22.53 | 12.68 | 34.46 | 22.65 | 12.54 | 34.96 |
| Minas Gerais | 22.85 | 12.62 | 35.18 | 22.80 | 12.83 | 34.80 | 22.81 | 12.59 | 35.22 |
| Paraná | 22.84 | 12.61 | 35.23 | 22.81 | 12.82 | 34.83 | 22.92 | 12.68 | 35.27 |
| Paraíba | 24.00 | 13.27 | 36.71 | 23.87 | 13.45 | 36.40 | 24.02 | 13.27 | 36.75 |
| Pará | 23.33 | 12.86 | 35.93 | 23.24 | 13.05 | 35.47 | 23.49 | 12.97 | 36.00 |
| Pernambuco | 23.23 | 12.85 | 35.70 | 23.12 | 13.00 | 35.26 | 23.40 | 12.95 | 35.93 |
| Piaui | 23.50 | 12.97 | 36.00 | 23.45 | 13.19 | 35.74 | 23.58 | 13.04 | 36.16 |
| Rio de Janeiro | 23.42 | 12.92 | 35.96 | 23.37 | 13.15 | 35.59 | 23.57 | 13.02 | 36.19 |
| Rio Grande do Norte | 22.89 | 12.67 | 35.16 | 22.84 | 12.89 | 34.84 | 23.14 | 12.80 | 35.58 |
| Rio Grande do Sul | 22.60 | 12.50 | 34.83 | 22.49 | 12.68 | 34.46 | 22.81 | 12.62 | 35.13 |
| Rondônia | 24.82 | 13.76 | 37.93 | 24.66 | 13.92 | 37.48 | 24.78 | 13.73 | 37.74 |
| Roraima | 24.08 | 13.30 | 37.01 | 24.03 | 13.55 | 36.61 | 24.18 | 13.37 | 36.96 |
| Santa Catarina | 23.04 | 12.70 | 35.50 | 22.90 | 12.90 | 35.03 | 23.14 | 12.78 | 35.64 |
| Sergipe | 22.91 | 12.66 | 35.31 | 22.84 | 12.86 | 34.86 | 23.12 | 12.77 | 35.52 |
| São Paulo | 22.83 | 12.63 | 35.19 | 22.79 | 12.83 | 34.78 | 23.14 | 12.79 | 35.62 |
| Tocantins | 23.27 | 12.84 | 35.84 | 23.17 | 13.03 | 35.41 | 23.42 | 12.92 | 36.02 |

SEV: Summary exposure value; *Age-standardized; U.I.: uncertainty interval.
